# Molecular characterization and pathological identification of a novel strain of delta papillomavirus-4 (bovine papillomavirus-2) in Egypt

**DOI:** 10.14202/vetworld.2021.2296-2305

**Published:** 2021-09-03

**Authors:** Rabab T. Hassanien, Mervat E. Hamdy, Sara M. Elnomrosy, Heba A. Hussein, Ahmed F. Afify, Fatma M. Darwish, Gehan Shehab, Rawhya Emran, Mervat I. I. Abd-El-Moniem, Ahmed R. Habashi, Hanan A. Fahmy, Essam M. Ibraheem, Momtaz A. Shahein, Mohamed Attya, Ali M.M. Abdelhakim, Naglaa M. Hagag

**Affiliations:** 1 Department of Virology Research, Animal Health Research Institute, Agriculture Research Center (ARC), 12618 Dokki, Giza, Egypt; 2 Genome Research Unit, Animal Health Research Institute, Agriculture Research Center (ARC), 12618 Dokki, Giza, Egypt; 3 Department of Pathology Research, Animal Health Research Institute, Agriculture Research Center (ARC), 12618 Dokki, Giza, Egypt; 4 Department of Biotechnology Research, Animal Health Research Institute, Agriculture Research Center (ARC), 12618 Dokki, Giza, Egypt; 5 General Organization of Veterinary Services, Dokki, Giza, Egypt.

**Keywords:** bovine papillomaviruses-2, first detection, histopathology, immunohistochemistry, phylogeny, transmission electron microscopy

## Abstract

**Background and Aim:**

Bovine papillomaviruses (BPV) are a heterogeneous group of oncoviruses, distributed globally, which produce major economic losses. In the current study, we compared the results of different diagnostic approaches and compared the strains identified in this study with previously characterized strains at local and international levels.

**Materials and Methods:**

Samples of skin warts were collected from five bovines with generalized papillomatosis from two Egyptian provinces, Menya and Ismailia, in 2020. Electron microscopy, molecular characterization, histopathological, and immunohistochemical examination were performed.

**Results:**

BPV was detected using electron microscopy in the collected samples. Using molecular characterization, BPV-2 was successfully identified for 1^st^ time in Egypt. The strain has 99.6% identity with the BPV-2 reference strains obtained from GenBank. These results were supported by histopathology and immunohistochemistry examination. Partial nucleotide sequences of the L1 gene were submitted to GenBank with accession numbers MW289843 and MW289844.

**Conclusion:**

BPV-2 was reported for 1^st^ time in the current study. The strain was identified grossly, microscopically, and pathologically and confirmed using molecular approaches. All results were consistent. The sequence analysis revealed that this strain has high sequence similarity to the reference Deltapapillomavirus-4, BPV-2 strains from Brazil and China.

## Introduction

Papillomaviruses (PVs) are a diverse group of oncogenic DNA viruses found in various species of mammals, as well as in some species of birds and reptiles [1,2]. PVs have been recognized in most domestic animals, including bovines (bovine PVs [BPV]), sheep (*Ovis aries* papillomavirus), goats (*Capra hircus* papillomavirus), equines (*Equus caballus* papillomavirus), canines (CPVs), domestic felines (*Felis domesticus* papillomavirus), and swine (*Sus scrofa* papillomavirus), as well as in humans (human papillomavirus). These viruses are thus of considerable veterinary and economic significance [2]. PVs are strictly species and site-specific and do not infect hosts other than their natural host, even under experimental conditions [1]. Infection of equids by BPV-1 and BPV-2 is the only known case of cross-species infection [3]. At least 53 genera in the family *Papillomaviridae*, including more than 200 forms of PV, have been identified to date. The Greek alphabet is used to classify genera ranging from *Alphapapillomavirus* to *Dyoiotapapillomavirus*, and the taxonomic nomenclature of PV types of animals is based on the scientific name of their host genus and species. Bovine papillomatosis is caused by BPV that belong to the *Papillomaviridae* family. The virion is small, icosahedral, non-enveloped, and about 55-60 nm in diameter, containing approximately 8 kbp of circular double-stranded DNA. It has one of the slowest evolutionary rates among viruses [1]. The genome consists of three major regions; the long control region and two regions of early and late genes encoding ORFs [4,5]. Currently, 28 forms of BPV have been characterized and grouped into five distinct genera: *Delta papillomavirus*, *Xi Papillomavirus*, *Epsilon papillomavirus, Dyoxipapillomavirus*, and *Dyokappappillomavirus*, which are associated with various pathological outcomes [6]. Each genus includes more than one type of BPV; *Delta papillomavirus* 4 (BPV 1, BPV 2, BPV 13, and BPV 14), *Xi Papillomavirus* (BPV 3, BPV 4, BPV 6, BPV 9, BPV 10, BPV 11, BPV 12, BPV 15, BPV17, and BPV 23), *Epsilon papillomavirus 1* (BPV5, and BPV 8), *Dyoxipapillomavirus 1* (BPV7, BPV 19, and BPV 21), and *Dyokappappillomavirus* genus (BPV16, and BPV18). Seven other BPV types, BPV20, BPV21, BPV 22, BPV 24, BPV 25, BPV26, and BPV 27, belong to unclassified genera [7-11].

BPV is a heterogeneous group of globally distributed epitheliotropic viruses. The virus favors the stratified squamous epithelium of warm-blooded animals [12]. Papillomavirus infection causes decreases in milk and meat production, poor hide value, and in extreme cases, mortality, and may therefore result in significant economic losses [13]. Cutaneous warts are most common in young animals and typically regress spontaneously without any scarring because of the immune response of the animals. The length of the infection is very variable, and recurrence is likely to occur from 1 month to 1 year [14]. As a preliminary diagnosis, the gross indication of the clinical lesions appearing on the skin is required, accompanied by histopathological examination, electron microscopy [15], or polymerase chain reaction (PCR) [7]. BPVs have been recorded in some Middle Eastern countries, such as Turkey [16], Iraq [9], and Egypt [17].

In the current study, we evaluated a range of diagnostic tools used for BPV, including electron microscopy, molecular identification, histopathological, and immunohistochemistry (IHC) techniques. We also performed a phylogenetic study using the Egyptian BPV strain and previously characterized BPV strains, global and local, to detect any evolutionary changes.

## Materials and Methods

### Ethical approval

This statement confirms that sampling for this research was performed as non-experimental ­clinical work with respecting the rules of the veterinary profession. All samples were collected by veterinarians. Sampling was performed strictly at the owner’s request. International, national, and\or institutional guidelines for sampling were followed with the approval of the local ethics committee on animal experimentation at the Animal Health Research Institute (AHRI), Agriculture Research Center, Egypt.

### Study period and location

Five cases of generalized papillomatosis were observed during the summer season (June, July, and August) of 2020 in private quarantines at Menya and Ismailia provinces in Egypt.

### Sample collection and preparation

**Figure-1:**
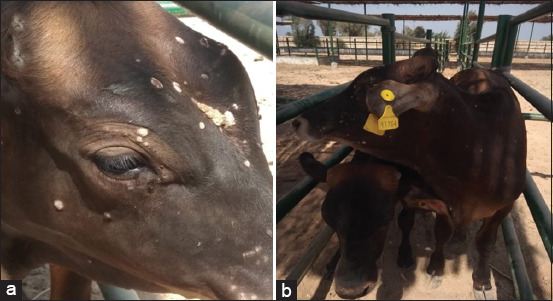
(a and b) Generalized papillomatosis (skin warts) of variable sizes distributed on different areas on body of suspected cases of bovine papillomavirus infection.

The cattle were suspected to be suffering from generalized papillomatosis with multiple papillomae (warts) of variable sizes ranging from 2 to 70 mm, disseminated on the face, head, neck, shoulders, and back. The warts of the head and face had a cauliflower-like appearance and were strongly attached to the dermis (Figures-1a and b). The preliminary diagnosis was made based on the clinical signs, since the structure of the skin papillomae was easily observed and identified. However, a few warts from each animal were surgically removed, transported in an icebox using biosecurity measures as specified by the World Organization for Animal Health [18] (transport of biological material), and submitted to the AHRI, to confirm the diagnosis.

For electron microscopy and molecular characterization; the warts were ground using sterile sand in 0.01 M phosphate-buffered saline (pH, 7.4) (Sigma-Aldrich, St Louis, Missouri, USA) to a final 10% (W/V) suspension. Tissue homogenate was centrifuged in a cooling centrifuge (Centurion Scientific, K3 Series, UK) at 3000 RPM for 15 min. The supernatant was then filtered and clarified using a 0.45 mm filter syringe (Millex AA syringe filter unit, Merck, USA). The filtrate was stored at −80°C for further analysis.

For histopathology and IHC; samples were prepared as detailed below.

### Transmission electron microscopy (TEM)

For TEM, two sample suspensions, one from each governorate, were dropped on carbon grids for 10 min, and then fixed using 3% phospho-tungstate (Sigma-Aldrich) for 10 min. The material was examined with the electron microscope (JEOL JEM 2100, Japan), and the images were captured using a Mega View III camera (Model S03U, EMSIS GmbH, Muenster, Germany) and using the software ITEM version E 23082007 (Olympus Soft Imaging Solutions, Hamburg, Germany), employing a voltage of 80 kV, a constant current of 1 A, and a total magnification of 60,000-120,000×. The examination was carried out at the EM unit, Central Research Laboratories, Faculty of Agriculture, Cairo University, Egypt [15].

### Molecular identification

#### DNA extraction and molecular diagnosis

Viral DNA was extracted from the samples using Easy-Pure Viral DNA/RNA Kits (Cat No: ER201) according to the manufacturer’s instructions. The extracted DNA was quantified using Spectrostar^Nano^ (BMG Labtech, Ortenberg, Germany).

#### Detection of different serotypes of BPV using real-time PCR

Extracted DNs were screened using real-time PCR to detect positive samples of BPV using BioIngenTech Bovine Papillomavirus (BPV) 13 Types-Real-Time DNA Kits (BioIngenTech, Concepción, Chile; Catalog no. PU-A003). Twenty microliters of total qPCR mix were prepared, using 10 mL of Universal qPCR master mix, 2 mL primer, probes, and internal control universal mix, 2 mL of DNA, and 6 mL of water. PCR was performed using the following cycling program; 95°C for 5 min, followed by 40 cycles of 95°C for 30 s, 60°C for 30 s, and 72°C for 30 s [19].

#### Detection of different serotypes of BPV using conventional PCR

Viral identification was performed based on the homology of the sequences of the BPVs, to reveal the genera of the BPVs, Xi papillomavirus, and Delta Epsilon papillomavirus (Delta-Epsilon), using two pairs of degenerated primers.

The Xi forward primer was (5′-TWYA ATAGDCCVTTTTGGAT-3′), and the reverse primer was (5′-TTMCG CCTACGCTTTGGCGC-3′). The Delta-Epsilon forward primer was (5′-CCAGAY TAYYTMAAAATGGC-3′), and the reverse primer was (5′-ATAAMKGCTAGCTTATAT TC-3′). These primers were used for the detection of the L1 ORF, resulting in 600 bp and 430 bp amplification products, respectively [20].

Another set of primers specific for a Partial L1 gene was also used. They included the FAP59-L1 gene forward primer (5′-TAACWGTIGGICAYCCWTATT-3′) and the FAP64-L1 gene reverse primer (5′-CCWATATCWVHCATITCICCATC-3′), which amplify a product of 484 bp [21].

Amplification was carried out in a 50 mL reaction volume containing 25 mL of COSMO PCR RED Master Mix (Cat. No. 1020300X), 2.5 mL of each primer set, 5 mL of the DNA sample, and nuclease-free water, with the thermal protocol: 95°C for 2 min, 95°C for 15 s, 55°C for 55 s, 72°C for 1 min, and 72°C for 10 min.

The PCR products were separated using electrophoresis on 1.5% agarose gel (AppliChem, Germany, GmbH). A gene ruler 100 bp DNA ladder (Fermentas, Thermo, Germany) was used to determine the fragment size. The gel was photographed using a gel documentation system (Alpha Innotech, Biometra) ™ and the data were analyzed using computer software (Alphamager™ gel imaging software) (https://www.biocompare.com/Product-Reviews/41059-AlphaImager-Gel-Imaging-System-From-Alpha-Innotech/).

### Sequence and phylogenetic characterization

DNA bands of the expected size were excised from the gel and purified using the QIAquick Gel Extraction Kit (Qiagen, Hilden, Germany), according to the manufacturer’s instructions. The purified PCR products were sequenced directly using ABI PRISM Big Dye Terminators v3.1Cycle Sequencing Kits (Applied Biosystems, Waltham, Massachusetts, USA). The products of the sequencing reactions were cleaned-up using a Centrisep purification kit (Applied Biosystems). The purified products were sequenced directly using an ABI PRISM3500genetic analyzer (Applied Biosystems).

BLAST^®^ (https://blast.ncbi.nlm.nih.gov/Blast.cgi) analysis was initially performed to check sequence identities [22]. A phylogenetic tree was constructed using the MegAlign module of the Laser gene DNA Star version 12.1 software (https://www.dnastar.com/), using the maximum likelihood method [23].

### Histopathology

Surgically obtained warts (tumors) were immediately immersed in 10% formalin at room temperature for at least 48 h before processing. Fixed specimens were dehydrated using graded alcohols and embedded in paraffin wax. Serial sections were cut 4 mm thick, stained with hematoxylin and eosin, and examined with a light microscope equipped with an ocular micrometer (Nikon Eclipse E600, Tokyo, Japan) [10].

### IHC

Neoplastic cell proliferation of bovine skin fibropapilloma was estimated in paraffin sections of skin tumor masses by the detection of P_53_ protein expression using avidin-biotin immunoperoxidase complex. The test procedures were performed following the manufacturer’s instructions. Tissue sections were incubated with a monoclonal antibody for P_53_ protein (Dakocorp, Carpentaria, Germany) and reagents required for the avidin-biotin complex (Vactastain ABC peroxidase kit). P_53_ expression was visualized using chromogen 3, 3 diaminobenzidine tetrahydrochloride (Sigma Aldrich).

## Results

### TEM

**Figure-2:**
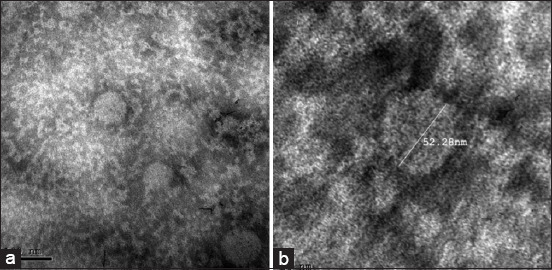
(a and b) Transmission electron microscopy of the negative-stained skin warts tissue containing papillomavirus particles, showing clusters of circular-shaped virus particles ranged from 55 to 70 nm in diameter at two captured scales; 50 and 100 nm with two different magnification 75000 and 120000×.

Examination of the negatively stained two samples using TEM showed a distinctive arrangement of BPV. Virus particles were observed to be circular, intact particles, ranging from approximately 55 to 70 nm in size (Figures-2a and b). Images were captured at 50 and 100 nm.

### Detection of different serotypes of BPV using real-time PCR

**Figure-3:**
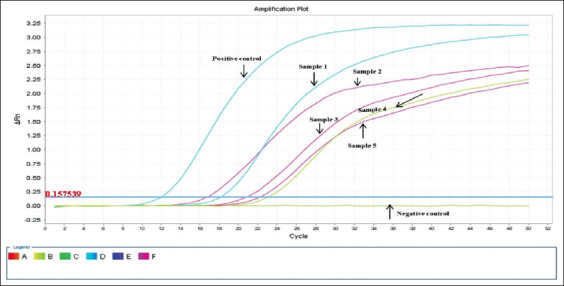
Real-time polymerase chain reaction (PCR) showed amplification plots based on threshold cycles (Ct) values in qPCR (one Step qPCR-real time ™ BPVDNA real time PCR kit) on DNA isolated from skin samples. Amplification curves had Ct values 12, 16.3, 18, 21, 22, and 23 for positive control and five tested samples, respectively.

The five samples examined tested positive with the real-time PCR kit, with amplification curves which had Ct values of 12, 16.3, 18, 21, 22, and 23 for the positive control and the five tested samples, respectively (Figure-3)

### Detection of different serotypes of BPV using conventional PCR

Using the Delta-Epsilon primers, the five samples examined were positive, with specific M.W. bands at 430 bp, but they were negative for the Xi primers. All samples were confirmed using FAP59-L1 gene primers with specific M.W. bands at 484 bp.

### Sequence and phylogenetic characterization

**Figure-4:**
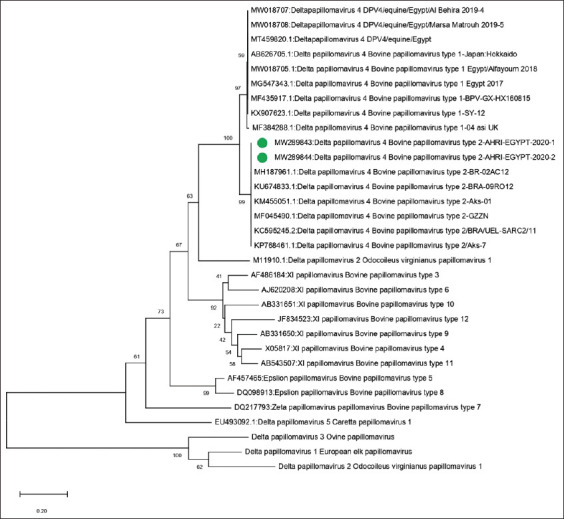
The phylogenetic tree created by MEGA 7 software (Pennsylvania state university, USA, www.megasoftware.net) using the maximum likelihood method. The confidence level of the neighbor-joining tree was evaluated by bootstrapping using 1000 replicates. The circles refer to the bovine papillomavirus -2 strains in our study, with their names and accession numbers.

The partial nucleotide sequences of the FAP59-L1 gene of two representative samples named Delta papilloma 4, BPV-2, AHRI-1-Egypt 2020 and Delta papilloma 4, BPV-2, AHRI-2-Egypt 2020, were submitted to GenBank with the accession numbers MW289843 and MW289844, respectively. Analysis of the sequence data revealed that our strains had 99.6% identity with Delta papillomavirus 4, the BPV-2 reference strain from Brazil (accession no: KC595245) and 98.6% identity to a Chinese strain (accession no: KP768461) (Figure-4 and Table-1).

**Table 1:** Percentage of nucleotide sequence identity between the strains of the current study (named BPV-AHRI-EGY-2020 strains) and reference strains published on GenBank based on partial nucleotide sequences of the FAP59- L1 gene.

S. No	Strains	1	2	3	4	5	6	7	8	9	10	11	12	13	14	15	16	17	18	19	20	21	22	23	24
1	MW289843:Delta papillomavirus 4 Bovine papillomavirus type 2-AHRI-EGYPT-2020-1	ID	100.0%	99.6%	98.6%	78.4%	78.4%	79.5%	79.5%	79.5%	46.7%	64.5%	59.0%	58.3%	55.6%	55.2%	55.6%	56.3%	56.3%	53.2%	53.2%	55.6%	36.8%	33.4%	34.8%
2	MW289844:Delta papillomavirus 4 Bovine papillomavirus type 2-AHRI-EGYPT-2020-2	100.0%	ID	99.6%	98.6%	78.4%	78.4%	79.5%	79.5%	79.5%	46.7%	64.5%	59.0%	58.3%	55.6%	55.2%	55.6%	56.3%	56.3%	53.2%	53.2%	55.6%	36.8%	33.4%	34.8%
3	KC595245.2:Delta papillomavirus 4 Bovine papillomavirus type 2/BRA/UEL-SARC2/11	99.6%	99.6%	ID	100.0%	78.4%	78.4%	79.5%	79.5%	79.5%	46.7%	64.5%	59.0%	58.3%	55.6%	55.2%	55.6%	56.3%	56.3%	53.2%	53.2%	55.6%	36.8%	33.4%	34.8%
4	KP768461.1:Delta papillomavirus 4 Bovine papillomavirus type 2/Aks-7	98.6%	98.6%	100.0%	ID	78.4%	78.4%	79.5%	79.5%	79.5%	46.7%	64.5%	59.0%	58.3%	55.6%	55.2%	55.6%	56.3%	56.3%	53.2%	53.2%	55.6%	36.8%	33.4%	34.8%
5	MG547343.1:Delta papillomavirus 4 Bovine papillomavirus type 1 Egypt 2017	78.4%	78.4%	78.4%	78.4%	ID	100.0%	97.6%	97.6%	97.6%	47.0%	62.7%	56.9%	55.9%	54.2%	52.9%	51.1%	57.3%	55.2%	50.1%	51.5%	53.2%	38.2%	34.8%	33.7%
6	MW018705.1:Delta papillomavirus 4 Bovine papillomavirus type 1 Egypt/Alfayoum_2018	78.4%	78.4%	78.4%	78.4%	100.0%	ID	97.6%	97.6%	97.6%	47.0%	62.7%	56.9%	55.9%	54.2%	52.9%	51.1%	57.3%	55.2%	50.1%	51.5%	53.2%	38.2%	34.8%	33.7%
7	MT459820.1:Deltapapillomavirus 4 DPV4/equine/Egypt	79.5%	79.5%	79.5%	79.5%	97.6%	97.6%	ID	100.0%	100.0%	46.7%	63.1%	56.6%	55.6%	54.6%	52.5%	50.8%	56.6%	54.9%	51.1%	51.5%	54.6%	37.8%	34.4%	34.1%
8	MW018707:Deltapapillomavirus 4 DPV4/equine/Egypt/Al Behira 2019-4	79.5%	79.5%	79.5%	79.5%	97.6%	97.6%	100.0%	ID	100.0%	46.7%	63.1%	56.6%	55.6%	54.6%	52.5%	50.8%	56.6%	54.9%	51.1%	51.5%	54.6%	37.8%	34.4%	34.1%
9	MW018708:Deltapapillomavirus 4 DPV4/equine/Egypt/Marsa Matrouh 2019-5	79.5%	79.5%	79.5%	79.5%	97.6%	97.6%	100.0%	100.0%	ID	46.7%	63.1%	56.6%	55.6%	54.6%	52.5%	50.8%	56.6%	54.9%	51.1%	51.5%	54.6%	37.8%	34.4%	34.1%
10	EU493092.1:Delta papillomavirus 5 Caretta papillomavirus 1	46.7%	46.7%	46.7%	46.7%	47.0%	47.0%	46.7%	46.7%	46.7%	ID	45.7%	50.1%	47.4%	47.9%	45.8%	45.5%	50.6%	46.9%	42.4%	43.1%	48.4%	31.9%	32.9%	35.0%
11	M11910.1:Delta papillomavirus 2 Odocoileus virginianus papillomavirus 1	64.5%	64.5%	64.5%	64.5%	62.7%	62.7%	63.1%	63.1%	63.1%	45.7%	ID	56.6%	52.9%	57.3%	52.2%	54.2%	55.9%	55.3%	53.7%	52.7%	51.5%	32.7%	33.4%	35.1%
12	AF486184:XI papillomavirus Bovine papillomavirus type 3	59.0%	59.0%	59.0%	59.0%	56.9%	56.9%	56.6%	56.6%	56.6%	50.1%	56.6%	ID	72.2%	67.2%	66.2%	61.7%	68.6%	63.4%	48.8%	51.8%	54.9%	32.4%	30.7%	33.4%
13	X05817:XI papillomavirus Bovine papillomavirus type 4	58.3%	58.3%	58.3%	58.3%	55.9%	55.9%	55.6%	55.6%	55.6%	47.4%	52.9%	72.2%	ID	65.5%	65.8%	63.1%	70.3%	66.5%	51.5%	53.2%	53.9%	33.4%	34.8%	35.1%
14	AJ620208:XI papillomavirus Bovine papillomavirus type 6	55.6%	55.6%	55.6%	55.6%	54.2%	54.2%	54.6%	54.6%	54.6%	47.9%	57.3%	67.2%	65.5%	ID	72.7%	70.9%	71.2%	65.0%	54.5%	55.9%	54.6%	36.2%	34.8%	35.5%
15	AB331650:XI papillomavirus Bovine papillomavirus type 9	55.2%	55.2%	55.2%	55.2%	52.9%	52.9%	52.5%	52.5%	52.5%	45.8%	52.2%	66.2%	65.8%	72.7%	ID	68.8%	77.1%	65.4%	52.9%	51.9%	54.2%	34.4%	34.1%	35.8%
16	AB331651:XI papillomavirus Bovine papillomavirus type 10	55.6%	55.6%	55.6%	55.6%	51.1%	51.1%	50.8%	50.8%	50.8%	45.5%	54.2%	61.7%	63.1%	70.9%	68.8%	ID	69.5%	63.3%	52.2%	53.9%	53.5%	33.1%	32.0%	31.7%
17	AB543507:XI papillomavirus Bovine papillomavirus type 11	56.3%	56.3%	56.3%	56.3%	57.3%	57.3%	56.6%	56.6%	56.6%	50.6%	55.9%	68.6%	70.3%	71.2%	77.1%	69.5%	ID	65.7%	51.5%	53.6%	54.9%	35.0%	32.9%	34.0%
18	JF834523:XI papillomavirus Bovine papillomavirus type 12	56.3%	56.3%	56.3%	56.3%	55.2%	55.2%	54.9%	54.9%	54.9%	46.9%	55.3%	63.4%	66.5%	65.0%	65.4%	63.3%	65.7%	ID	56.5%	54.8%	53.2%	32.0%	32.4%	35.1%
19	AF457465:Epslion papillomavirus Bovine papillomavirus type 5	53.2%	53.2%	53.2%	53.2%	50.1%	50.1%	51.1%	51.1%	51.1%	42.4%	53.7%	48.8%	51.5%	54.5%	52.9%	52.2%	51.5%	56.5%	ID	78.4%	50.1%	36.5%	32.4%	36.5%
20	DQ098913:Epslion papillomavirus Bovine papillomavirus type 8	53.2%	53.2%	53.2%	53.2%	51.5%	51.5%	51.5%	51.5%	51.5%	43.1%	52.7%	51.8%	53.2%	55.9%	51.9%	53.9%	53.6%	54.8%	78.4%	ID	48.4%	35.1%	31.0%	35.1%
21	DQ217793:Zeta papillomavirus Bovine papillomavirus type 7	55.6%	55.6%	55.6%	55.6%	53.2%	53.2%	54.6%	54.6%	54.6%	48.4%	51.5%	54.9%	53.9%	54.6%	54.2%	53.5%	54.9%	53.2%	50.1%	48.4%	ID	34.8%	35.8%	35.8%
22	Delta papillomavirus 1 European elk papillomavirus	36.8%	36.8%	36.8%	36.8%	38.2%	38.2%	37.8%	37.8%	37.8%	31.9%	32.7%	32.4%	33.4%	36.2%	34.4%	33.1%	35.0%	32.0%	36.5%	35.1%	34.8%	ID	71.0%	67.1%
23	Delta papillomavirus 2 Odocoileus virginianus papillomavirus 1	33.4%	33.4%	33.4%	33.4%	34.8%	34.8%	34.4%	34.4%	34.4%	32.9%	33.4%	30.7%	34.8%	34.8%	34.1%	32.0%	32.9%	32.4%	32.4%	31.0%	35.8%	71.0%	ID	68.9%
24	Delta papillomavirus 3 Ovine papillomavirus	34.8%	34.8%	34.8%	34.8%	33.7%	33.7%	34.1%	34.1%	34.1%	35.0%	35.1%	33.4%	35.1%	35.5%	35.8%	31.7%	34.0%	35.1%	36.5%	35.1%	35.8%	67.1%	68.9%	ID

### Histopathology

**Figure-5:**
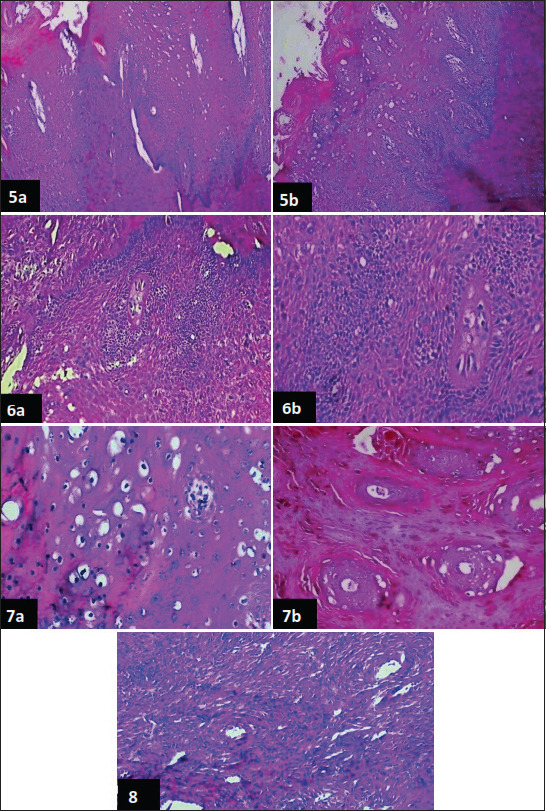
(a) Skin showing variable degrees of acanthosis; hyperkeratosis; Parakeratosis; Koilcytosis associated with down growth of rete ridges, (b) Notice the hyperplasia of the basal cells layer. The acanthotic prickle cell layers showing marked vacuolation and numerous inflammatory foci (H&E stain 200×), **Figure-6:** (a and b) skin showing marked hyperplasia of the basal cell layer; these hyperplastic cells showing pleomorphism; round or ovoid nuclei and scanty cytoplasm and these are accompanied with mitotic activity (H&E stain, A: X200-B: 400×), **Figure-7:** (a) Skin showing Koilcytosis where the keratinocytes of the upper stratum spinosum are swollen; have clear cytoplasm (perinuclear halo and pyknotic nucleus), (b) Cells nest formed by koilocyte in between parakeratotic skin (H&E stain, a: X200, b: 400×), **Figure-8:** Skin showing g dermal fibroblastic proliferation; the proliferating cells are large fibroblast arranged in haphazard Whorls; sometimes around blood vessels (H&E stain 400×).

The histopathological examination of bovine skin warts of different cases revealed variable degrees of acanthosis and epidermal interdigitation that projected outwardly or inwardly, forming rete ridges. These were associated with marked hyper and/or parakeratosis (Figures-5a and b). The most pronounced changes were vacuolation, especially in the upper layer of the stratum spinosum, which extended in some cases to form inflammatory cysts. Focal areas of necrosis were also seen. The cases examined showed sub-epidermal congestion, round cell infiltration, and the formation of fibrous tissue.

The most obvious histopathological change observed in this study was hyperplasia of the basal cell layer of the skin. The hyperplastic cells appeared closely packed and pleomorphic, with basophilic round or ovoid nuclei, and a small amount of cytoplasm (Figures-6a and b). Mitotic activity was detected within numerous cells.

Koilcytosis was seen microscopically in many fields. However, the keratinocytes of the upper stratum appeared swollen, with clear cytoplasmic halos and pyknotic nuclei (Figure-7a), these koilocytes were arranged in clusters (cell nests) in between the parakeratotic skin (Figure-7b).

Histopathological examination showed features of fibropapilloma additional to acanthosis, hyperkeratosis, and down-growth of rete ridges: The dermal layer proliferated, with large fibroblasts. The proliferating fibroblastic cells were arranged in haphazard whorls and around blood vessels (Figure-8). In some cases, these fibroblastic proliferations were minimal, but were associated with slight acanthosis and hyperkeratosis.

### IHC

**Figure-9:**
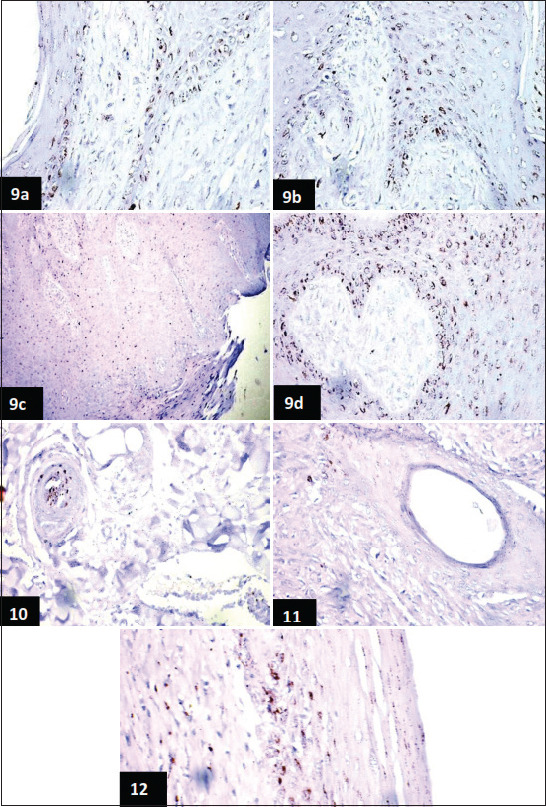
Different microscopic images for skin fibropapilloma caused by BPV examined with immunohistochemistry through detection of P35 protein expression, images were taken at 600× magnification power. (a) Section of bovine skin of fibropapilloma revealing widespread immunopositivity of P53 protein among the epithelial cells of the epidermis as well as in the basal and para-basal cells of the rete ridges (Avidin biotin complex immunoperoxidase counter stain by Mayer’s. (b) Section of bovine skin in fibropapilloma showing spread immunopositivity of P53 among the proliferated cells of the epidermis and dermal papillae (Avidin-biotin complex immunoperoxidase staining 600×). (c and d) Section of bovine skin of fibropapilloma revealing strong immunopositivity of P53 within the cellular nucleus and cytoplasm of the proliferated cells of epidermis and the dermal papillae (Avidin biotin complex immunoperoxidase, c (200×) and d (600×), **Figure-10:** Section of bovine skin of fibropapilloma revealing marked expression of P53 in the lining vascular endothelial cells and within the wall of the blood vessels (Avidin biotin complex immunoperoxidase 600×), **Figure-11:** Section of bovine skin of fibropapilloma showing positive expression of P53 in the epithelial cells lining the outer root of hair follicles, in addition to a granular reaction in the inner one (Avidin biotin complex immunoperoxidase 600×), **Figure-12:** Section of bovine skin of fibropapilloma revealing strong positive expression of P53 in the cells of the epidermis and in the proliferated fibroblast cells of the dermis (Avidin biotin complex immunoperoxidase 600×).

Neoplastic cell proliferation of bovine skin ­fibropapilloma was evaluated using a P53 protein expression marker. In the present study, the epithelial constituents of the fibropapilloma showed variable positive expression of the P53 protein in the cellular nucleus and/or within the cytoplasm of the stratum corneum, granulosum, spinosum, and basal layer cells. Strong positive expression of P53 protein was also seen in the basal and parabasal cells of the rete ridges (Figures-9a-d). Our studies also showed intense positive expression of this protein within the vascular endothelial cells of both the epidermal and dermal layers (Figure-10). However, a weak positive reaction was seen in the cytoplasm of the epithelial cells lining the outer root of some hair follicles, and a granular reaction was observed in the inner root (Figure-11). Strong expression of P35 was also observed in the proliferated fibroblast cells in the dermis (Figure-12).

## Discussion

BPV is an infectious disease of major economic importance all over the world. Little is known about BPV in Egypt. Although BPV is a self-limiting disease [5], the clinical cases in our study suffered from long-lasting multiple tumors which did not seem to regress for months, as previously observed by Araldi *et al*. [15]. On gross examination, the tumors exhibited variable-sized dense sessile or flat circumscribed masses, pedunculated, cornified, wrinkled surface, and lack of hair. These features are referred to morphologically as a typical form of bovine papillomatosis [1,20,24,25]. This disease is caused by epitheliotropic oncogenic viruses, and is characterized by multiple benign tumors that can regress spontaneously or progress to malignant neoplasms [26].

It has been reported that BPV affects only females [20]. However, our study identified the presence of multiple papillomae in male calves, a finding in agreement with that of Babu *et al*. [25]. Calves in this study infected with PBV were aged 1-2 years, and thus were matched very well with the subjects of the other study [20]. However, several researchers have reported BPV infection in both young and adult animals aged from 6 months to 10 years [25,27,28].

While Vitiello *et al*. [20] reported lesions on only the teats of heifers, the clinical cases in our study were found to have warts on different parts of the body, including the face, head, base of horns, neck, dewlap, shoulders, and back. These findings were in accordance with previous research [1,7,24,25,28]. In the present study, BPV was detected using electron microscopy. The virus particles appeared as intact, circular particles, ranging in size between 55 and 70 nm. Similar results have previously been recorded in several studies [1,17,27,29-31].

For further confirmation of the TEM results, molecular identification was performed on tested samples, as previously suggested [11,32]. All tested samples were positive with both qPCR and conventional PCR assays, results which matched well with those of other researchers [6,33-36]. The conventional PCR protocol using degenerate primers was successful in detecting a novel strain of BPV in Egypt [21,37,38].

The sequencing and phylogenetic analysis results showed that the strains in the current study (accession numbers MW289843 and MW289844) showed a high degree of homology with the international Delta papillomavirus 4, and BPV-2 strains. The similarity was 99.6% with the Brazilian and 98.6% with the Chinese BPV-2 reference strains from GenBank (accession numbers KC595245 and KP768461, respectively). The strains obtained in this study were not related to previously characterized Egyptian isolates collected in 2017, 2018, and 2019 [17,36]. The sequence identity was 78.4% with the Egyptian strains of 2017 and 2018 (Delta papillomavirus 4 Egypt 2017 (accession no: MG547343) and Delta papillomavirus 4 Egypt/Alfayoum 2018 (accession no: MW018705), as the 2017 and 2018 strains were closely related, and were genotyped as BPV-1. When comparing our strains with the strains isolated in Egypt in 2019, the results revealed 79.5% identity with Delta papillomavirus 4 DPV4/equine/Egypt/Al Behira 2019-4 (accession no: MW018707) and Delta papillomavirus 4 DPV4/equine/Egypt/Marsa Matrouh 2019-5 (accession no: MW018708). These results highlight the novelty of this study, and confirm the detection of the Delta papilloma 4, BPV-2 strain for the 1^st^ time in Egypt.

Histologically, our study showed a variable degree of cellular proliferation, involving both the epidermal and dermal layers, associated with epidermal interdigitation and exophytic mass formation; similar results have previously been recorded [14,15]. This phenomenon occurred due to the effect of oncogenic protein E_5_ which interacts with different proteins, promoting the loss of adhesiveness of the cells and cell polarity, and contributing to invasiveness [14,39]. The current study showed hyper and/or parakeratosis of the superficial susceptible keratinocytes. This picture was in accordance with the finding of Betz [40], who reported that the assembly of the virus within the proliferated cells increased the production of keratin granules that provide physical protection to the virus.

One of the most important findings was vacuolation of the proliferated cells, with the formation of koilocytes. This proliferation may contribute to cell fragility and the release of virions [15]. Nassour *et al*. [41] found that the cytopathic effect of ­oncogenic proteins was responsible for koilocyte formation. Sub-epidermal congestion, round cell infiltration, and fibrous tissue formation have previously been reported in different studies [14,42].

The stimulation of cell proliferation by BPV induces mitotic activity in the proliferated cells, particularly in the stratum basal, as the virus induces DNA damage and genomic instability [38,43].

Cell transformation and differentiation were related to the oncogenic protein E_5_ in the E region of the virus [15]. The oncogenic protein E_7_ contributes to cell cycle deregulation and induces DNA breaks [26,42].

Fibropapillomae were recorded in the current study. They may turn to the oncogenic proteins which are responsible for the fibrotropism [44,45]. E_5_ oncoproteins can bind and activate platelet-derived growth facto b on the surface of pericytes, stimulating neoangiogenesis, which represents a very important picture for the tumor [46].

Various cellular markers were used for the identification of tumor proliferation. One approach is microscopic estimation of nuclear antigen, which is related to cell division and growth, using IHC tools [14]. In the present study, the estimation of neoplastic cell proliferation of bovine skin fibropapilloma was carried out using the P_53_ protein expression marker. Strong positive expression of P_53_ protein was seen in some cellular nuclei, and in the cytoplasm of the epithelial cells of the stratum corneum, granular, spinous, parabasal, and basal layers of the skin epidermis. Similar findings have been obtained by other studies [14,47]. Our results were also compatible with a previous study, that detected cytoplasmic and perinuclear P_53_ expression in equine sarcoid induced by PBV [26].

The observation of positive P_53_ protein expression in the present study may be due to the impairment of the P_53_ mechanisms caused by the neoplastic transformation effects of the oncogenic bovine papillomavirus. The impaired mechanisms cause a transitory increase of P_53_ protein, leading to its accumulation in the cellular cytoplasm [14]. Few studies have stressed the function of BPV oncogenes in the development of cancer by the disruption of cellular mechanisms and the facilitation of neoplastic transformation [2,34,48].

The sequestration of wild type of P_53_ into the cell cytoplasm is associated with DNA destruction, leading to cell cycle arrest and apoptosis [49]. Our IHC findings were also supported by those of Al-Salihi *et al*. [14], who found irregular positive expression of P_53_ protein in the vascular endothelial cells and mesenchymal cells of fibropapilloma.

## Conclusion

Gross identification, TEM examination, full molecular characterization, histopathological, and IHC examination were successfully carried out, and the results were consistent with those of previous studies. Genotyping and phylogenetic analysis played an important role in the identification of the BPV-2 (*Deltapapillomavirus-4*) strain for the 1^st^ time in Egypt. Further broad, statistically designed, molecular-based, and cohort studies are needed to systematically evaluate the epidemiology of BPV, and to detect any other types of papillomavirus in Egypt.

## Authors’ Contributions

MAS and MA: Designed the study, AFA: Collected the samples. AMMA and ARH: Collected the data of the samples. FMD, GS, RE, and EMI: Performed the pathology and histopathology for the collected samples. RTH, HAH, and MIIA: Carried out the samples preparation and electron microscope identification. NMH, HAF, MEH, and SME: Performed the molecular diagnosis (Real time PCR, Conventional PCR, sequencing, and phylogenetic analysis). MAS, EMI, HAF, and ARH: Made the final revision. All authors: Wrote the original draft, discussed the result, and approved the final manuscript.
